# Coaching patients in the use of decision and communication aids: RE-AIM evaluation of a patient support program

**DOI:** 10.1186/s12913-015-0872-6

**Published:** 2015-05-28

**Authors:** Jeff Belkora, Shelley Volz, Meredith Loth, Alexandra Teng, Margot Zarin-Pass, Dan Moore, Laura Esserman

**Affiliations:** Philip R. Lee Institute For Health Policy Studies, University of California, San Francisco, 3333, California St, Suite 265, San Francisco, CA 94118 USA; Helen Diller Family Comprehensive Cancer Center, University of California, San Francisco, 1600 Divisadero Street, San Francisco, CA 94115 USA; Carol Franc Buck Breast Care Center, University of California, San Francisco, 1600 Divisadero Street, 2nd Floor, San Francisco, CA 94115 USA

**Keywords:** Decision aids, Communication aids, Breast cancer, RE-AIM, Patient support, Coaching, Audio-recording, Question List, Consultation summary

## Abstract

**Background:**

Decision aids educate patients about treatment options and outcomes. Communication aids include question lists, consultation summaries, and audio-recordings. In efficacy studies, decision aids increased patient knowledge, while communication aids increased patient question-asking and information recall. Starting in 2004, we trained successive cohorts of post-baccalaureate, pre-medical interns to coach patients in the use of decision and communication aids at our university-based breast cancer clinic.

**Methods:**

From July 2005 through June 2012, we used the RE-AIM framework to measure Reach, Effectiveness, Adoption, Implementation and Maintenance of our interventions.

**Results:**

1. Reach: Over the study period, our program sent a total of 5,153 decision aids and directly administered 2,004 communication aids. In the most recent program year (2012), out of 1,524 eligible patient appointments, we successfully contacted 1,212 (80 %); coached 1,110 (73 %) in the self-administered use of decision and communication aids; sent 958 (63 %) decision aids; and directly administered communication aids for 419 (27 %) patients. In a 2010 survey, coached patients reported self-administering one or more communication aids in 81 % of visits
2. Effectiveness: In our pre-post comparisons, decision aids were associated with increased patient knowledge and decreased decisional conflict. Communication aids were associated with increased self-efficacy and number of questions; and with high ratings of patient preparedness and satisfaction
3. Adoption: Among visitors sent decision aids, 82 % of survey respondents reviewed some or all; among those administered communication aids, 86 % reviewed one or more after the visit
4. Implementation: Through continuous quality adaptations, we increased the proportion of available staff time used for patient support (i.e. exploitation of workforce capacity) from 29 % in 2005 to 84 % in 2012
5. Maintenance: The main barrier to sustainability was the cost of paid intern labor. We addressed this by testing a service learning model in which student interns work as program coaches in exchange for academic credit rather than salary. The feasibility test succeeded, and we are now expanding the use of unpaid interns.

**Conclusion:**

We have sustained a clinic-wide implementation of decision and communication aids through a novel staffing model that uses paid and unpaid student interns as coaches.

## Background

Approximately 230,000 women every year are diagnosed with breast cancer in the United States [1]. Participating in breast cancer treatment decisions is often difficult for patients because the diagnosis is a shock and throws many patients into cognitive and emotional overload. Patient participation in decision making is important though, because common treatments (surgery, radiation, chemotherapy, hormone therapy) differ in impact on survival, recurrence, and quality of life. A needs assessment found that breast cancer patients are exposed to “too much, too little, or conflicting information” while waiting to see a specialist. Then, at the appointment, patients often “freeze up and forget to ask questions.” When physicians do answer patient questions, the information “goes in one ear and out the other.” [2] Evidence based interventions such as decision aids and communication aids have emerged to help patients meet these decision making and communication needs [3–5].

Decision aids are print or audio-visual materials designed to educate patients about treatment options and outcomes. In efficacy studies decision aids have been shown to increase patient knowledge and decrease decisional conflict [5–8], among other benefits. Communication aids, which include question lists, consultation summaries and audio-recordings, increase patient question-asking during consultations and information recall afterwards [9–16]. Decision and communication aids have been shown to be beneficial and are therefore ready for broader dissemination and implementation. However, a recent systematic review found only 17 studies reporting on implementations of either decision or communication aids [17].

We are the first to combine decision and communication aids in a single multi-component support program. In 2005, with support from a philanthropic foundation, we integrated decision and communication aids into our university-based breast cancer clinic using staff serving in paid internship positions. We designed our program, initially known as Decision Services (now the Patient Support Corps), using program theory [18] and the theory of diffusion of innovations [19], as described in a case study [20]. We used the RE-AIM framework (Reach, Effectiveness, Adoption, Implementation, Maintenance), to monitor and inform continuous improvements to our implementation [21, 22]. In response to calls for more research on the translation of evidence-based decision support interventions into practice [5, 17], we are now reporting on our findings. This report addresses the need for health services research on implementation and dissemination, addressing calls in the United Kingdom for more T3 research [23] and in the USA for more T2 research [24]. We report here on the reach, effectiveness, adoption, implementation and maintenance as measured during the first seven years of our implementation.

## Methods

From July 2005 through June 2012, we measured Reach, Effectiveness, Adoption, Implementation, and Maintenance of decision communication aids using the methods and measures outlined below. In order to minimize patient burden, we staggered the collection of data over different sample time frames.

### Study setting, population, sample, design, timing

The study took place in the Breast Care Center at the University of California, San Francisco. The Breast Care Center is a high volume clinic providing multidisciplinary care in a National Cancer Institute-designated Comprehensive Cancer Center. The underlying population consisted of all the patients who made “new patient” appointments to see Breast Care Center specialists from July 2005 to June 2012. During this time period, the population was majority white (65 %), college-educated (85 %) and insured (60 % private insurance). We attempted to contact all these patients to offer coaching along with decision and communication aids. We solicited survey responses from those patients who accepted our materials or services, administering items by email or paper before and after they received decision aids; before and after question-listing sessions; before and after medical consultations; and four weeks after the first consultation. We changed the surveys every program year between 2005 and 2012 in order to measure different outcomes without increasing the burden of responding. We also analyzed records logged by staff in our program database. See Table 1 for sample time frames and collection dates and Table 2 for study participant demographics.

**Table 1:** Sample time frames and collection dates

T-1	T-2	T-3	T-4	T-5
Patient completes survey prior to viewing Decision Aid	Patient completes survey after viewing Decision Aid	Coach completes survey after question-listing session with patient	Patient completes survey waiting to see specialist	Patient is sent survey 4 weeks post-visit with specialist
Data collected between Nov. 2005 and Oct. 2008		Data collected between July 2009 and June 2012	Data collected between July 2009 and June 2012	Data collected between July 2009 and June 2012
1553 DAs with surveys distributed to 1098 patients, 549 completed surveys returned, 35 % RR		1016 staff surveys completed for 1032 question-listing sessions, 98 % RR	822 surveys collected from 1124 office visits, 73 % RR	741 surveys completed from 1871 invitations, 40 % RR

**Table 2:** Demographics of patients served with coaching or decision or communication aids (abstracted from electronic medical record)

Race			
	White	2026	59 %
	Missing data	571	17 %
	Asian	448	13 %
	Other	240	7 %
	African-American	131	4 %
	Total	3416	100 %
Hispanic Ethnicity			
	No	2304	67 %
	Missing data	925	27 %
	Yes	187	5 %
	Total	3416	100 %
Language Interpretation			
	Cantonese	8	
	Mandarin	6	
	Russian	7	
	Spanish	23	
	Tagalog	1	
	Total		
Insurance			
	Missing data	1816	53 %
	Managed care (HMO, PPO)	1078	32 %
	Medicare	294	9 %
	Medi-Cal	102	3 %
	Self Pay	126	4 %
	Total	3416	100 %
Education*			
	8th grade or less	8	1 %
	Some high school	11	1 %
	Graduated High School	58	7 %
	Some college	184	22 %
	Bachelor’s degree	227	28 %
	Some graduate school	81	10 %
	Master’s degree	159	19 %
	Ph.D., M.D., J.D., or other	71	9 %
	Missing data	23	3 %
	Total	822	100 %

*Education data collected at T4; other demographics captured from clinic schedule system

We obtained a waiver of written consent and ethics approval from the UCSF Committee on Human Research to abstract and de-identify our program records for research analysis and reporting purposes. In this manuscript we report on data not previously published, while also summarizing data reported in earlier publications [8, 14, 15, 20, 25–29].

### Interventions –coaching in the use of decision and communication aids

#### Workforce

During the study period, our program personnel consisted of one part-time director (author JB), one part-time coordinator (author SV), and a revolving staff of interns (including authors ML, AT, and MZP). Each program year began with the arrival of new interns, as the prior year interns concluded their post-baccalaureate year and went on to medical school or graduate programs in science or public health. Each year, the director led a two day training workshop, teaching interns how to administer decision aids, elicit and document patient questions, and make notes and recordings while remaining neutral and non-directive. During a two-week overlap period, the new interns apprenticed with the departing interns, practicing under the supervision of experienced peers. The director and coordinator also supervised weekly case review meetings, provided interns with a detailed program manual, fielded intern queries by phone and email, and audited program records to assure quality.

Once trained, interns called patients two to three weeks prior to their consultation appointments to encourage the use of decision and communication aids (described below) and alerted patients verbally as well as by mail or email of other available resources such as our hospital’s Cancer Resource Center; patient health library; and online resources approved by Breast Care Center clinicians. Interns were each assigned a list targeting patients who had upcoming “new” or “new 60 minute” appointments to see a surgeon or medical oncologist and tasked with calling them a minimum of two times. Interns used interpreters when making phone calls to patients with language preferences indicated in the scheduling system. If patients did not respond to calls or reply to voicemails, interns sent them a template email describing and encouraging the use of decision and communication aids. Interns either enclosed these resources as links, or the email referred patients to resources being sent by mail, or available at the Cancer Resource Center.

### Decision aids

In addition to calling and corresponding with our patients by email, program interns also routinely mailed one or more applicable decision aids to newly diagnosed patients before their decision-making appointments with doctors at the Breast Care Center. The five available decision aids, provided by the Informed Medical Decisions Foundation covered options and outcomes for treating ductal carcinoma in situ, early stage, and metastatic breast cancer.

### Identifying and ordering applicable decision aids

We trained schedulers, practice assistances, call center staff and interns to identify, based on clinic notes and conversations with the patient, which decision aid(s) matched the patient diagnosis. Having found one or more applicable decision aids, the staff then typed a special code into the patient appointment record in our scheduling system. By looking for the appearance of this code in the scheduling system, our program coordinator then knew to mail out the decision aid(s) prior to the patient visit, or include a link to the decision aid(s) in an email.

### Communication aids

Our interns coached patients to self-administer communication aids by suggesting patients (1) make a list of their questions; (2) bring a note-taker; and (3) make an audio-recording of the visit. The interns sent interested patients a prompt sheet by mail or email [30]. Interns also offered to accompany patients to their visits and administer the communication aids for them. In that scenario, an intern interviewed the patient for 20 to 60 minutes by telephone a few days before the clinic visit, and wrote down a word-processed list of the patient’s questions. The intern sent the question list to the physician in advance of the appointment; brought a laptop and digital audio-recorder to the visit to take notes and make a recording; and sent a summary of the notes and a compact disc copy of the audio-recording to the patient and physician.

During the question-listing session conducted by telephone, the intern followed a neutral, non-directive protocol called SLCT which stands for Scribing (writing down) the patient top-of-mind questions without interrupting; then Laddering or asking for elaboration on each of the initial questions; then Checking or administering a prompt sheet of additional topics; and finally Triaging the question list into a single page [31]. The interns used their training in summarizing and paraphrasing to write down the patient questions succinctly, while staying true to the patient intended meaning; and likewise summarized and paraphrased the physician’s advice and information in a neutral manner. Interns listened to the consultation audio recordings when necessary to assure the accuracy of their notes.

Due to the constrained availability of interns, we rationed our in-person services according to perceived patient need. Specifically, during the outreach calls, our interns placed patients on a waitlist and assigned each a priority level based on the intern’s assessment of need as well as the patient’s self-reported need for the service. Typically this dialogue generated a high priority level to patients who were unaccompanied, had limited English proficiency, were especially distressed or cognitively impaired, or who simply stated they would like to be considered high priority for their own reasons, disclosed or undisclosed.

**Fig. 1:**
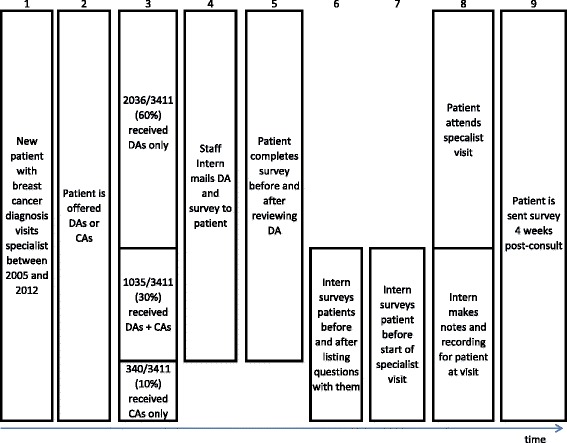
Sequence and timing of program interventions and surveys of effectiveness. This figure represents the sequence of interventions and surveys used during the study period. We used the survey time points to collect different data during different program years. See manuscript text for details. Abbreviations: DA = Decision Aid; CA = Communication Aid

See Fig. 1 for an overview of the intervention timeline.

### Standardized measures and instruments for effectiveness

We asked patients who received decision and communication aids to complete surveys, respond to verbal prompts, or complete online surveys at five different time points as shown in Fig. 1. We used validated instruments with known psychometric properties to measure standard outcomes of decision support, summarized below, following the Ottawa Decision Support Framework [32, 33]. For study-specific outcomes, we created custom instruments of our own [34], which we briefly describe in each corresponding Results section.

To measure effectiveness in decision aids we asked patients to respond to questions measuring knowledge and decisional conflict. Respondents answered 3 to 6 multiple choice and open-ended condition-specific questions regarding treatment options for early-stage breast cancer decisions including surgery, reconstruction, and adjuvant therapy [8, 35, 36]. The knowledge items demonstrated good re-test reliability (intraclass correlation coefficient = 0.70) and content validity (discriminated between providers and patients – mean difference 35 %, p < 0.001) [37].

We also measured decisional conflict, defined as a state of uncertainty about the course of action to take [38]. The Decisional Conflict (DCS) scale is a 16-item Likert scale with a test-retest correlation coefficient of 0.81, and alpha coefficients ranging from 0.78 to 0.92 [38]. The version we used employed a response scale of 1 to 5 with 1 indicating the least amount of decisional conflict and 5 indicating the most. We asked respondents to respond to three of the five subscales (Informed, Values Clarity, and Uncertainty) from the Decisional Conflict Scale for a total of 9 questions before and after reviewing the decision aids.

To measure the effectiveness of our question-listing intervention, we used patient-reported self-efficacy. The Decision Self-Efficacy scale is an 11-item Likert scale measuring patients’ confidence in their ability to be informed and involved in treatment decisions [39]. The version we used employed a response scale of 1 to 5 with 1 indicating minimum confidence and 5 indicating maximum confidence. This scale was previously found to have acceptable psychometric properties and was sensitive to decision support interventions [40, 41].

To measure preparedness to make decisions, we used relevant items from the Preparation for Decision Making Scale [42]. Validation studies found this 10-item Likert scale had acceptable psychometric properties and was sensitive to decision support interventions [41]. To minimize patient response burden, we selected the most relevant items and adapted the wording to reflect our question-listing intervention: Did the question-listing session (and videos/booklets, if you received any) help you think about how involved you want to be in this discussion? Did the question-listing session (and videos/booklets, if you received any) help you identify the questions you want to ask? Did the question-listing session (and videos/booklets, if you received any) prepare you to talk to your doctor about what matters most to you? The response options were “1 – not at all,” “2 – a little,” “3 – somewhat,” “4 – quite a bit,” and “5 – a great deal.”

## Results

### Reach

Reach is defined as the absolute number, proportion, and representativeness of individuals who participate in a given initiative [22]. In the most recent program year of the study (2011-2012), out of 1,524 eligible patient appointments, we successfully contacted 1,212 (80 %); coached 1,110 (73 %) in the self-administered use of communication aids; sent 958 (63 %) decision aids; and provided staff-administered communication aids for 419 (27 %). Table 3 shows how our reach expanded over time, growing from 54 % to 73 % for patients coached in the self-administered use of communication aids; 25 % to 63 % for decision aids sent; and 17 % to 26 % for communication aids administered by staff. Over the study period (2005 to 2012), our staff sent a grand total of 5,153 decision aids and directly administered 2,004 communication aids.

**Table 3:** Annual reach of program interventions

Reach	2005-2006 PY	2006-2007 PY	2007-2008 PY	2008-2009 PY	2009-2010 PY	2010-2011 PY	2011-2012 PY
New patient clinic appointments	821	1377	1051	1331	1355	1416	1524
Patients called and reached (% of new appointments)	NA*	NA*	NA*	NA*	727 (92 %; 54 %)	875 (85 %; 62 %)	1212 (80 %)
Patients coached in use of decision and communication aids (% of called and reached; % of new appointments)	NA*	NA*	NA*	NA*	727 (92 %; 54 %)	875 (85 %; 62 %)	1110 (92 %, 73 %)
Decision aids sent (% of new appointments)	208 (25 %)	389 (28 %)	648 (62 %)	936 (70 %)	1027 (75 %)	987 (70 %)	958 (63 %)
Staff-administered communication aids (% of new appointments; % of staff capacity)	142 (17 %; 29 %)	208 (15 %; 47 %)	245 (23 %; 50 %)	285 (21 %; 65 %)	348 (26 %; 73 %)	357 (25 %; 72 %)	419 (27 %; 84 %)

*We did not document the number of patients called and reached until the creation of our online program database in May 2009. That is also the year in which we began coaching patients to self-administer communication aids, i.e. list questions, take notes, and make recordings by themselves. Abbreviations: PY = Program Year; NA = Not Available

In cases when our staff directly administered communication aids, we were confident that our efforts resulted in these interventions reaching patients. However, we were also interested in the reach of communication aids when we simply coached patients to self-administer them. Between January and September 2010, we surveyed 195 consecutive coached patients and received 82 responses (42 %). Four out of five (63/78 or 81 %) responded that they wrote a question list in response to our prompting, although only one of every four (14/61 or 23 %) said they showed it to their doctor. Two-thirds (51/77 or 66 %) said they brought a note-taker, but only 16/79 (20 %) reported making audio recordings. We have described these findings in greater detail in a prior publication and summarize them here for completeness [28].

### Effectiveness

#### Decision aids

Of 1,553 surveys distributed between November 2005 and October 2008 to assess the effect of decision aids, we received 549 usable completed surveys (35 % response rate computed as survey responses divided by surveys sent). For the four early stage decision aids, our raw results showed 580 correct responses to 1275 questions prior to reviewing the decision aid (45 % correct) as compared to 938 correct responses to the same 1,275 questions after (74 % correct), a statistically significant increase in knowledge (p < 0.001). Overall respondents reported mean decisional conflict of 2.61 prior to viewing the decision aid, falling to 2.09 afterwards, a statistically significant decrease in decisional conflict (p < 0.001). Respondents rated their satisfaction with these decision aids at a mean of 4.2 out of a maximum of 5 [8].

Survey respondents found the decision aids helpful. Representative comments included:

- “Your program helped me to focus and resulted in me changing treatment options. I know I made the right decision for me. I now sleep at night.”
- “The video [Early Stage Surgery] was very helpful; I wish I could have had this info as reference when I had my first cancer occurrence.”
- “It was helpful for me to see photos [Reconstruction] of women who have chosen different options after their mastectomy. I also appreciate the candid interviews of each of the women.”

We have described these findings in greater detail in a prior publication and summarize them here for completeness [8].

#### Communication aids

**Fig. 2:**
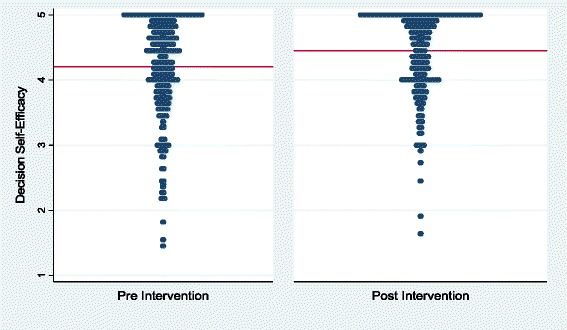
Distribution of decision self-efficacy scores (line denotes mean)

We evaluated decision self-efficacy before and after question-listing sessions over an 18-month period (January 2007 through June 2008). We collected surveys from 321/362 sessions (89 % response rate) resulting in 242 matching pairs with responses for at least 10 of the 11 items both before and after. The mean decision self-efficacy before the question-listing session was 4.20 (95 % confidence level 4.10 to 4.29) rising to 4.45 (95 % confidence level 4.37 to 4.53) afterwards, a statistically significant increase of 0.25 (paired t-test p < 0.001). See Fig. 2.

**Fig. 3:**
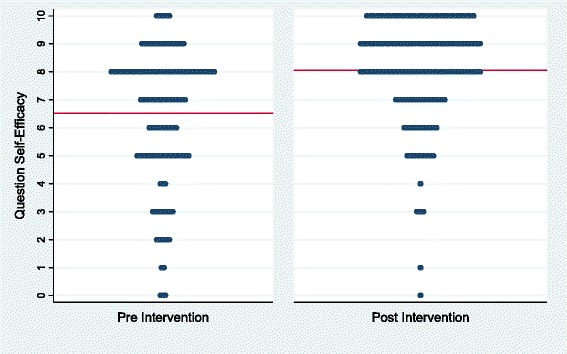
Distribution of question self-efficacy scores (line denotes mean)

To reduce patient burden, we subsequently surveyed patients about a single item measure, “I know what questions to ask my doctor,” which we felt captured the essence of our question-listing program. Between May and December 2009, we collected 161 surveys before and after 209 question-listing sessions (77 % response rate) resulting in 137 matched pairs. The mean before the question-listing session was 6.6 (95 % confidence interval 6.2 to 7.0) and after was 8.0 (95 % confidence interval 7.7 to 8.4). This shows a statistically significant increase of 1.4 (paired t-test p < 0.001). See Fig. 3.

**Fig. 4:**
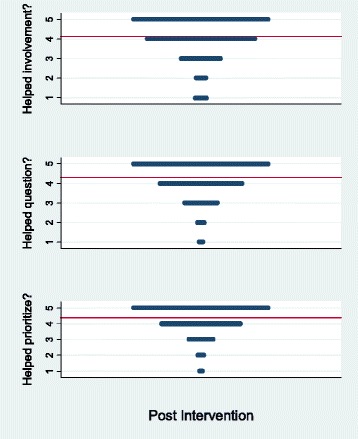
Preparation for decision making (line denotes mean)

From July 2009 through June 2012 patients completed 416 questionnaires that included three items from the Preparation for Decision Making Scale while waiting in the exam room to see their specialists for 557 appointments (75 % response rate). Most (297/378 or 79 %) responded the question-listing session had helped them “4 – quite a bit” or “5 – a great deal” think about how involved they wanted to be in the discussion (mean 4.17). Most (283/374 or 75 %) responded the question-listing session had helped them “4 – quite a bit” or “5 – a great deal” to identify the questions they wanted to ask (mean 4.02). Most (309/377 or 82 %) responded the question-listing session had helped them “4 – quite a bit” or “5 – a great deal” prepare them to talk to their doctor about what matters most to them (mean 4.25). See Fig. 4.

**Fig. 5:**
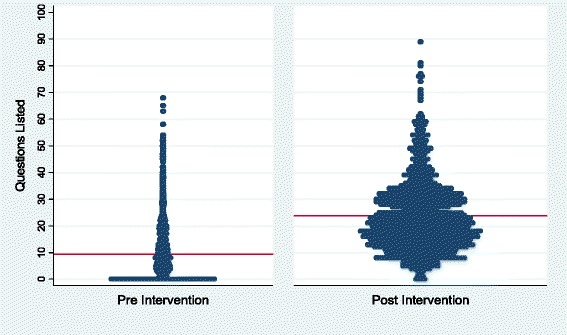
Distribution of number of questions listed (line denotes mean)

To measure the impact of the pre-visit question-listing intervention, we tabulated the number of questions patients emailed to us before our question-listing sessions or dictated to us over the phone for 36 months (July 2009 through June 2012). We collected data before and after 1,016 of 1,032 question-listing sessions (98 % response rate) resulting in 1,001 matched pairs. The mean number of questions before the question-listing session was 10 (95 % confidence interval 8.9 to 10.3) and after was 24 (95 % confidence interval 22.9 to 24.6). This shows a statistically significant increase of 14 (paired t-test p < 0.001; 95 % confidence interval 13.4 to 14.8). See Fig. 5.

**Fig. 6:**
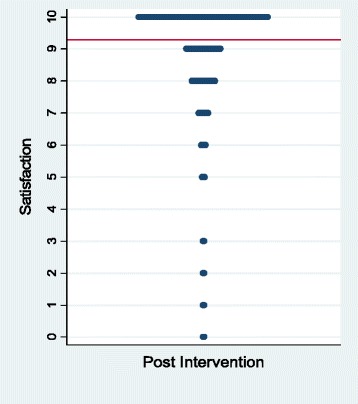
Satisfaction with question-listing and decision aids (line denotes mean)

From July 2009 through June 2012 we asked how satisfied patients had been with their pre-visit interventions immediately before seeing their providers. We collected 822 responses from 1,124 patients (73 % response rate). On a scale from zero to 10 the mean satisfaction rating was 9.3 See Fig. 6.

Regarding program effectiveness for staff, respondents to our survey of intern alumni (21/47 or 45 %) reported that their participation in Decision Services increased their perceived medical competencies across the board, while making the biggest contribution to respondent growth in systems-based practice and patient care. One respondent wrote, “Decision Services helped me actually learn the art of listening through practice, patience, and silence.” Another wrote, “After working for Decision Services, I think I was maximizing my potential in compassion and propriety in working with patients. This carried with me through medical school.” We previously reported on this intern survey in the literature [29].

### Adoption

#### Directly administered communication aids

We defined patient adoption as the level of patients’ acceptance, use of, satisfaction with, and willingness to recommend to others, our program interventions. From July 2009 through June 2012 there were 4,295 new visitors with new patient appointments, whom we monitored regarding adoption of direct assistance with communication aids. We spoke with 3,034 (71 %) of the 4,295 we tried to contact. We called during business hours and left at least two voice mail messages encouraging patients to call us back. When we had an email address on record for the patient, our second voicemail indicated we would be sending an email describing available resources and providing our contact information. More than half of those new visitors reached by telephone (1,675/3,034 or 55 %) accepted an intern offer to directly administer communication aids. We placed these on our waitlist and ranked them according to urgency of need for our services. One in three of the new visitors we spoke with (1,037/3,034 or 34 %) declined, often citing the accompaniment of a family member or friend to take notes. The remaining 11 % (322/3,034) were not eligible, because they didn’t know their diagnosis, or the consultation was to confirm a diagnosis, or they did not have breast cancer.

A total of 55 % of new patient visitors initially accepted our offer of direct assistance (1,675/4,295) from July 2009 through June 2012. We delivered direct assistance to 26 % of all new patient visitors (1,124/4,295), representing 67 % of those on the waitlist (1,124/1,675).

Of the new patients who initially accepted, 2 % (33/1,675) canceled their appointments, 7 % (117/1,675) changed their minds, and we lacked capacity to serve the remaining 24 % (401/1,675).

From July 2009 through June 2012, four weeks after their consultations, we asked visitors who had received communication aids to tell us which communication aids they had reviewed, shared with anyone, and would recommend to others. We collected 489 responses from 978 patients (50 % response rate) who had received communication aids. Seventy percent of respondents (249/358) indicated they had reviewed the question-list since the appointment, 60 % (220/367) had listened to the recording, and 86 % (297/344) had reviewed the summary since the appointment. Other results showed high recommendation rates for the question list, consultation summary, and consultation recording (see Table 4).

**Table 4:** Patient behaviors and attitudes regarding decision and communication aids after the visit

Decision and communication aids (DAs and CAs) July 2009 through June 2012 (surveys = 1812, responses = 741, 41 % response rate)								
	Received	Reviewed		Shared		Recommend		Satisfaction
	Total	n	%	n	%	n	%	DA Only surveys (252 responses), mean = 8.3
Decision Aid -- Booklet	1195	980	82 %	254	32 %	496	63 %	
Decision Aid -- Video	1195	792	66 %	219	28 %	468	59 %	
Question List	358	249	70 %	203	57 %	322	90 %	DA and CA surveys (489 responses), mean = 9.1
Consultation Recording	367	220	60 %	151	41 %	310	84 %	
Consultation Summary	344	297	86 %	216	63 %	312	91 %	

#### Decision aids

On the same four-week follow-up survey we asked all visitors who had received decision aids to report on how much of each they had reviewed, if they had shared them with anyone else, and if they would recommend them to others. We sent a total of 1,812 survey invitations to patients who had received any decision or communication aids between July 2009 and June 2012. The 741 respondents (41 %) had received a total of 1,195 decision aids. Of the 1,195 decision aids received, patients had reviewed “all” or “some” of 792/1195 (66 %) of the videos and had reviewed “all” or “some” of 980/1195 (82 %) of the booklets (see Table 4). Representative patient comments included:

- “I have reviewed the Summary notes several times, and was glad to have the recording to more easily recall what was discussed.”
- “I think it is wonderful and incredibly helpful to call patients and offer to accompany them to their consultation. In addition, the person I talked with recommended I bring a tape recorder and I now do so for almost all appointments. This is a valuable service that supports patients in the process of making difficult decisions and helps patients develop skills to promote their own health.”
- “After receiving the services, especially the notes from the Oncology Doctor visit, I read through the extensive, well organized notes [Intern] transcribed. I was incredibly surprised that I had only remembered about one-third or less of what the doctor said. I have referred back to these notes many times. This is an invaluable service to any patient.”
- “I really appreciated the consultation recording. Even though I haven’t felt the need to review it again, it gave me a very secure feeling that everything I discussed with Dr. [redacted] was being recorded so I did not have to worry about whether we (I and my friend) were taking good and complete notes. If I had wondered about any aspect of the visit, I knew I could go back and review it at any time. The consultation summary was extremely helpful, and one reason I have not gone back to review the actual recording.”

#### Physicians

Regarding adoption by physicians, we found universal cooperation among physicians, in the sense that all 22 agreed a priori to allow the use of decision and communication aids, whether administered by patients or staff. One physician joining the practice expressed reservations about being recorded, but agreed to try it for a month, and ultimately agreed to continue. Physicians were also generally collaborative in incorporating the decision and communication aids, and interns, into the consultation, for example by referring to the question list or repeating complex information for the note-taker to capture, or endorsing the use of decision and communication aids to put patients at ease. We reviewed intern process notes and found many examples of physician collaboration, such as:

- “He let the patient know that he had reviewed her questions and also used it [the question list] to go over her choices”
- “[MD] said he had read through the consultation plan and that I was getting down everything on the recorder and in my notes.”

These and other details are available from our qualitative analysis of intern process notes, published in the literature [27].

### Implementation

At the setting level, implementation refers to our fidelity to the various elements of a program protocol [22]. A major success factor in our implementation was the fact that the Breast Care Center leadership was willing to subsidize the participation of their staff in this job enrichment program by donating one day per week of each intern’s time. We wanted to make sure the staff was maximally engaged in obtaining the patient interaction experience we promised them. This led us to measure exploitation of staff capacity, or the proportion of available staff time used for direct patient support in our program. To quantify staff capacity we calculated the number of patients our interns could potentially accompany each month and compared it to the actual number of patients actually accompanied that month.

Over the first program year we exploited 142/496 (29 %) of our staff capacity. By 2011-2012, we had improved our exploitation of staff capacity to 84 % (419/500). See Table 3 to see the trajectory of workforce capacity exploitation. Overall, our most important innovation was to design an outbound calling program whereby our interns call patients and coach them, rather than wait for patients to self-refer or count on schedulers to refer them [8, 25], .

### Maintenance

Starting in 2004, the Informed Medical Decisions Foundation provided materials (decision aids) and funding to UCSF to support our program as a demonstration project, exploring the feasibility of integrating evidence-based decision and communication aids into practice. These external funds supported program design and implementation activities, along with data collection, analysis, and reporting. Upon expiration of external funding after the 2012 program year, the Breast Care Center continued its support of the program interns and took over 50 % funding of the program coordinator (approximately $45,000 including fringe benefits). We interpreted this commitment of resources as evidence of institutionalization at the Breast Care Center. The Breast Care Center also committed annual funds in the amounts of $3,000 for the program database, and $700 for mailing the decision aids. We therefore estimate the program’s maintenance costs at approximately $150,000 per year for paid interns, a coordinator, and supplies.

Over the course of our program history, UCSF patients outside the Breast Care Center also expressed interest in accessing our program. Administrators and clinical leaders endorsed the idea of expanding our program, but told us they did not have sufficient resources to pay interns. To address this barrier to further institutionalization, we obtained a grant from an innovation fund at our institution to explore using part-time unpaid interns alongside full-time paid interns.

Based on our findings, we implemented an affiliation agreement with the University of California, Berkeley nearby. The affiliation agreement specifies the legal, privacy, and risk management provisions required to safely and effectively deploy students in our clinical setting. We then demonstrated, in a small scale implementation, the feasibility, acceptability, and effectiveness of using students alongside our interns, inside as well as outside the Breast Care Center [43]. Two UC Berkeley students received academic credit for this service learning internship, delivering the question-listing, note-taking and audio recording services to six patients with appropriate fidelity to our program design.

We are therefore deploying unpaid interns alongside our paid interns in the Breast Care Center and using unpaid interns as a workforce to expand to other units. A hospital auxiliary foundation has provided a grant of $30,000 to launch the Patient Support Corps and we have trained 12 UC Berkeley student interns to support patients in the fertility preservation, gynecologic oncology, radiation oncology, urology, head and neck, colorectal, melanoma, orthopaedic, and spine clinics.

## Discussion

### Interpretation of results and connections to the literature

#### Reach

Our program has evolved to be distinct from others in our use of interns to call all of our clinic patients and coach them in the use of both decision and communication aids. Other programs have reported systematic distribution of decision aids alone, through staff or schedule-based requisitioning systems [44, 45]. Some have evaluated coaching in response to inbound calls [46, 47], including in combination with the distribution of decision aids [48]. We noted a finding in the literature that broad mailings could be associated with inappropriate materials in 20 % of cases [49]. When in doubt, rather than taking a best guess at the appropriate decision aid and sending it, the interns now tell patients to ask their provider which program might be best for them at the consultation.

Our reach of communication aids directly administered by program staff has stabilized at 1 in 4 due to our limits in capacity and the challenges of synchronizing intern availability with patient visits. We are encouraged that 81 % of respondents to our survey reported self-administering one or more communication aids based on our prompting. Implementation studies of question prompting, which invite patients to circle frequently asked questions and add their own via a sheet distributed by clinic personnel in person or by mail, found similar reach as our staff coaching patients to self-administer our prompt sheet [50, 51].

### Effectiveness

In our implementation, we found similar effectiveness as predicted by systematic reviews of randomized controlled trials of decision and communication aids [5, 41, 52]. The decision aids were associated with increased knowledge, consistent with findings in a systematic review confined to breast cancer [53]. Communication aids were associated with increased self-efficacy, increased number of questions, and high levels of satisfaction and ability to access information for recall purposes, also consistent with prior efficacy studies [54–57]. We found higher numbers of questions (mean 24) than were reported in the intervention arms in prior studies of question-prompting interventions (means ranged from 6-15) [3, 55, 58, 59].

### Adoption

Our results for patient viewing of decision aids, at 68 % (videos) to 82 % (booklets), were higher than the few other reports in the literature. Viewing rates have been reported at 25 % [49], and 38 % to 44 % [60] for decision aids. We attribute our higher rates to the fact that many of our patients reviewed the decision aids in preparation for a scheduled pre-consultation question-listing session with an intern. In essence, we gave patients a homework assignment with a deadline, and used the results of their decision aid review in a functional task, question-listing, which benefited them.

Listening rates in our study were equal to or lower, at 60 %, than in efficacy studies of audio-recordings. Under efficacy study conditions, patients have reported listening at rates of 60 % [4, 61], 75 % [56] and 89 % [58]. Patients in those studies may have been more likely to listen as a result of their study participation, whereas our patients did not know that they would be asked about their listening habits. Some patients wrote in follow-up survey comments that they appreciate having the recordings as a safety net, whether or not they listen to them.

### Implementation

Our primary concern about implementation has been to fully exploit the capacity of our paid interns. After we switched to outreach by interns, our capacity exploitation increased to acceptable levels that would sustain sponsor and staff interest (now 84 %). We also initiated a waitlist inspired by queuing theory in optimization [62]. This waitlist allowed us to synchronize intern availability with patient demand, according to the priority of patient needs.

### Maintenance

We launched our program with support from an external Foundation, which no longer provides funding. The Breast Care Center now sustains the program from its discretionary funds. In order to expand our program affordably, we are deploying student interns alongside paid interns. The student interns obtain academic credit for the service learning experience of coaching patients in the use of communication aids. We call this initiative the Patient Support Corps and are replicating it at other clinics and educational institutions.

### Study quality

The strengths of this study are that it reports on the only sustained implementation integrating coaching and decision and communication aids. We report on our translation of evidence-based decision and communication aids after evaluating the five RE-AIM dimensions of our program. We staggered the data collection for Effectiveness into separate phases so as to minimize the response burden on any given patient.

While RE-AIM is a well-accepted framework for implementation research, it emphasizes external validity, or the potential relevance of findings to real-world settings, over internal validity, or the rigor of inferences within the study. Indeed, this study has all the limitations of any pragmatic field evaluation, including relatively low internal validity for our inferences, as we lacked random assignment and control groups. We also experienced relatively low response rates for surveys administered online, by mail, or by telephone, outside of our in-clinic interactions with patients. For in-clinic surveys, we had high response rates but could have experienced agreement bias as the same staff often administered both interventions and surveys.

## Conclusions

We conclude that our sustained implementation of coaching with decision and communication aids has successfully translated evidence-based support strategies into practice. Our support program now has broad reach. We have seen the effectiveness predicted by prior randomized controlled trials, in terms of patient knowledge, decisional conflict, preparation for decision-making, satisfaction, self-efficacy, number of questions, and access to information for recall purposes. Patients have adopted the decision and communication aids, reviewing them and sharing them with others. We have adhered to the implementation plan for our interns, offering them the promised job enrichment featuring weekly patient interaction.

We have successfully integrated our program into the clinic workflow, minimizing clinic burden while enriching patient experiences. We have found satisfaction from all parties involved, including patients, interns, and physicians. We have maintained our implementation for over 7 years, with plans to sustain the program with students as well as interns.

We connected over 4,500 individuals with support for making high stakes, high stress decisions. We introduced 22 physicians and dozens of residents, fellows, nurses, and other health care specialists to these concepts and turned them into advocates for decision support. We trained 84 paid staff interns and 20 unpaid student interns participate in a patient-centered high-touch supportive service. Our model is evidence that this sort of program can be successfully integrated into specialty care and will contribute to patients making informed decisions with their attending physicians. For those sites that lack resources to pay coaches, our findings suggest that unpaid student interns can gain academic credit and experience while effectively delivering evidence-based decision and communication aids.

## Abbreviations

CA: Communication aid
DA: Decision aid
DCIS: Ductal carcinoma In Situ
NA: Not available
PY: Program year
RE-AIM: Reach, effectiveness, adoption, implementation, maintenance
SCOPED: Situation, choices, objectives, people, evaluation, decision
SLCT: Scribe, ladder, check, triage
